# Vegetative Propagation Preserves Genomic Diversity and Informs Translocation Strategies in a Rare Clonal Plant

**DOI:** 10.1111/eva.70299

**Published:** 2026-07-18

**Authors:** Rob Massatti, Susan Bainbridge, Stella M. Copeland, Carter Crouch, Trevor M. Faske, Erik P. Hamerlynck, Brandon Palmer, Carla M. Roybal

**Affiliations:** ^1^ Landscape Stewardship Collective Flagstaff Arizona USA; ^2^ U.S. Geological Survey Southwest Biological Science Center Flagstaff Arizona USA; ^3^ USDA‐Agricultural Research Service Eastern Oregon Agricultural Research Center Burns Oregon USA; ^4^ International Crane Foundation Rockport Texas USA; ^5^ Oregon Desert Land Trust Burns Oregon USA

**Keywords:** clonal plants, genetic monitoring, plant translocation, population genomics, vegetative propagation

## Abstract

Conservation translocations are widely used to increase population size and redundancy, yet their genetic consequences are often uncertain, particularly for clonal species with unknown rates of sexual reproduction. Propagation through asexual reproduction is frequently employed in these systems, but its effectiveness for preserving genomic diversity remains poorly evaluated. We used genome‐wide single nucleotide polymorphism (SNP) data to assess the genetic outcomes of translocation efforts in *Pleuropogon oregonus* , a critically endangered grass endemic to eastern Oregon, USA. We quantified genetic diversity, relatedness, and population structure across natural and introduced sites and used coalescent simulations to infer the divergence history between disjunct populations. Genetic analyses identified two divergent lineages corresponding to northern and southern regions, with divergence predating the last glacial period and limited subsequent gene flow. Within regions, natural populations exhibited high clonality but retained genetically distinct individuals. Introductions that used vegetative propagules from the northern region maintained heterozygosity and allelic diversity comparable to sources and captured multiple distinct genotypes, including alleles likely originating from an unsampled source, thereby increasing population redundancy. In contrast, the introduction derived from a low‐diversity, southern region source reflected similarly limited clonal diversity. Our results demonstrate that vegetative propagation can effectively preserve genomic diversity in clonal species when propagules are sampled representatively, but that evolutionary history may inform sourcing and mixing decisions. More broadly, this study provides an empirical framework for integrating genomic data into conservation translocations, highlighting conditions under which vegetative propagation maintains evolutionary potential and when it may pose risks to long‐term persistence.

## 1 Introduction

Conservation translocations are increasingly implemented to prevent extinction, restore populations, and increase redundancy across landscapes, yet their genetic consequences are often uncertain. This uncertainty is particularly acute for species with limited or unknown rates of sexual reproduction, for which vegetative propagation is frequently used to generate plant material for introduction. Although translocations can enhance species viability by increasing population size, restoring gene flow, and reducing extinction risk (Albrecht and Edwards 2020; Bragg et al. 2021; Van Rossum and Le Pajolec 2021), outcomes depend critically on how genetic variation is captured and distributed among introduced populations. Even when outcrossing rates are low, inadequate representation of genetic diversity or inappropriate mixing of divergent lineages may compromise fitness and long‐term persistence (Edmands 2007; Frankham et al. 2011). Despite widespread implementation of translocations, there remains limited empirical evaluation of when commonly used propagation strategies preserve evolutionary diversity and support effective management.

The need to understand the genetic consequences of translocation is especially pressing given ongoing declines in plant biodiversity. Globally, many vascular plant taxa are at risk of extinction (Lughadha et al. 2020; Bachman et al. 2024), making them among the most threatened taxonomic groups (Shivanna 2019). Declines in plant biodiversity impact human society (Dubey et al. 2007; Giam et al. 2010) and other species that depend on plants and the ecosystems they inhabit (Haddad et al. 2009; Wan et al. 2020). The primary drivers of decreased diversity are agriculture and extraction of biological resources (Lughadha et al. 2020), disturbances that are compounded by the increasing rate of climate change (Oliver and Morecroft 2014; Santos et al. 2021) and invasive species spread (Gilbert and Levine 2013; Downey and Richardson 2016). In addition, habitat loss and fragmentation have reduced many rare or declining plants to small, isolated, and genetically depauperate populations threatened by low fitness, high genetic load, and low capacity for resilience or adaptation (Aguilar et al. 2006, 2019; Angeloni et al. 2011). These factors contribute directly and synergistically to an increasing extinction debt (Lughadha et al. 2020). While proactive strategies, such as the protection of habitat, may reduce extirpations and extinctions, the pervasive impact of invasive species, climate change, and the legacy of habitat alteration and fragmentation often necessitate active interventions such as translocation (Zimmer et al. 2019).

Rare plants may be formerly widespread species reduced to small, isolated, relict populations due to habitat degradation or loss (e.g., Kaye et al. 1997; Lesica et al. 2016), paleoendemic taxa that persist today as remnants of lineages that were historically more widespread (Soto‐Trejo et al. 2024), or species that are inherently rare due to intrinsic biological limitations such as narrow niche space or limited population sizes (Wamelink et al. 2014). Either through habitat reduction or loss of connectivity, species of all types of rarity may be impacted by loss of gene flow, genetic diversity, and inbreeding (Angeloni et al. 2011; Arenas et al. 2012; Schlaepfer et al. 2018; Aguilar et al. 2019). Plant translocation, which includes the introduction of plants into habitat that they previously did not occupy, reintroduction of plants into habitat from which they are extirpated, and augmentation of existing populations, is increasingly implemented to bolster the viability and resilience of rare or declining species (Silcock et al. 2019; Zimmer et al. 2019; Diallo et al. 2021; Fenu et al. 2023; D'Agostino et al. 2024). Translocations may increase species viability by creating redundant populations and increasing population size. They may also help reverse some genetic consequences of fragmentation and population decline by restoring connectivity, reducing genetic drift, and maintaining genetic diversity. In some cases, translocations can be used to reestablish populations in areas where a species has been extirpated (Albrecht and Edwards 2020; Bragg et al. 2021; Van Rossum and Le Pajolec 2021).

Translocation activities may be risky due to interactions among genotypes or between genotypes and the environments they experience. When sexual reproduction occurs, augmentation of existing populations using genetically divergent material may increase vulnerability through loss of locally adapted alleles and/or reduced fitness due to outbreeding depression (Edmands 2007; Frankham et al. 2011; Bowles et al. 2015; Barmentlo et al. 2018), including loss of extant genotypes through genetic swamping (Morrison and Molofsky 2000; Lenormand 2002). Alternatively, augmentation could result in sterile progeny due to the introduction of incompatible cytotypes, including intraspecific variation in chromosome number, chromosome translocations, or chromosome inversions (Severns and Liston 2008; Kramer et al. 2018). Moreover, individuals established during introduction or reintroduction management efforts may fail in the short or long term due to maladaptation (Derry et al. 2019) and frequently exhibit reduced genetic diversity relative to their propagule source because of founder effects arising from limited sampling of natural populations or propagation techniques (Lauterbach et al. 2012; Whitlock et al. 2016). Low genetic diversity can have immediate negative effects on population growth and fitness (Williams 2001), as well as long‐term effects on resilience and evolutionary potential (Sgrò et al. 2011). For clonal species with limited seed production, however, low effective population size or limited sexual recruitment may not necessarily indicate short‐term demographic failure if persistent genets continue to produce viable ramets, though limited recombination may constrain longer‐term adaptive potential. Therefore, assessing potential risk and success requires understanding population structure, reproductive mode, and the natural and imposed factors that mediate structure and gene flow.

Here, we use genome‐wide single nucleotide polymorphism (SNP) data to evaluate the genetic consequences of vegetative propagation in *Pleuropogon oregonus* (Poaceae), a rare, clonal graminoid endemic to eastern Oregon, USA. This species occupies two disjunct regions and has been the focus of long‐term introduction efforts using vegetative propagules, providing a unique opportunity to assess how this strategy preserves genetic diversity and informs sourcing decisions. Specifically, we (1) quantify genetic diversity, relatedness, and population structure within and among individuals at natural and introduced *P. oregonus* sites, (2) use coalescent modeling to infer divergence history, migration rates, and population sizes to evaluate whether mixing individuals among regions may be appropriate, and (3) compare genetic patterns between natural and introduced sites to test whether vegetative propagation adequately captures genetic diversity and increases population redundancy. By linking evolutionary history and contemporary genetic patterns to management outcomes, this study provides an empirical framework for evaluating when vegetative propagation is likely to maintain evolutionary potential and support effective conservation translocations, with broader relevance for wetland and riparian restoration programs that rely on clonal plant material.

## 2 Methods

### 2.1 Study System

**FIGURE 1:**
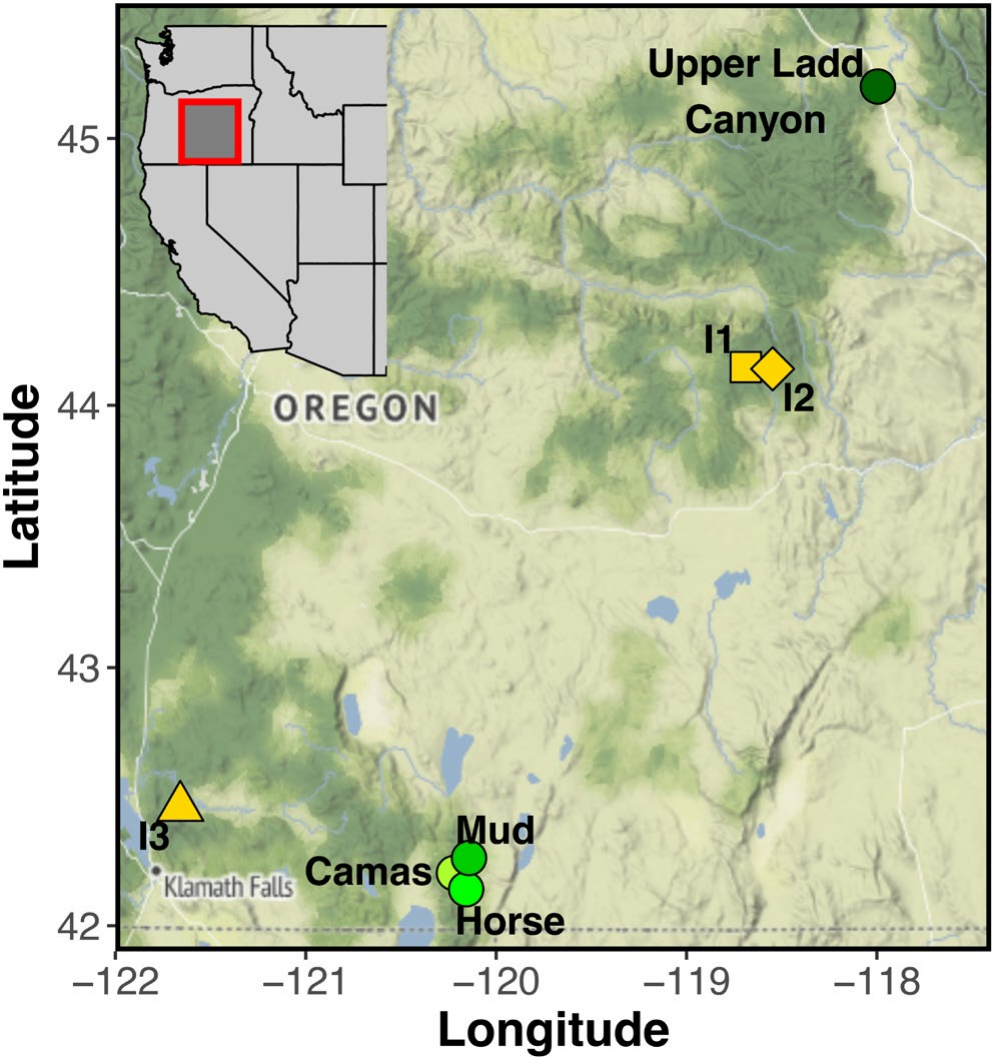
Locations of natural (green circles) and introduced (yellow shapes) sites for *Pleuropogon oregonus* in Oregon. Colors and shapes of symbols representing sampling sites are consistent with Figures 2 and 3.The North population is composed of upper Ladd Canyon, I1, and I2. The South population is composed of Camas Creek, Horse Prairie, Mud Creek, and I3. Basemap source: Stamen Terrain; map tiles © Stamen Design, CC BY 4.0; data © OpenStreetMap contributors, ODbL.

*Pleuropogon oregonus* Chase (Poaceae: Pooideae) is a rhizomatous perennial grass endemic to eastern Oregon. The genus contains four congeneric species in North America: three occupy wetlands west of the Sierra Nevada/Cascade mountain ranges in northern California, Oregon, and/or Washington, while one occurs primarily in arctic regions. The closest species geographically to *P. oregonus* is *Pleuropogon refractus* (A. Gray) Benth. ex Vasey. *Pleuropogon oregonus* is one of the rarest grasses in North America; by 1936, only three herbarium collections were known from two widely disjunct sites approximately 235 km apart. Between 1936 and 1979, no further collections or observations were recorded, and the species was presumed extinct (But et al. 1985). After rediscovery in 1979, *P. oregonus* is still only known from two disjunct areas: a cluster of approximately four sites in Union County, Oregon (hereafter “North”) and a cluster of approximately five sites in Lake County, Oregon (hereafter “South”; Figure 1). The North and South sites are separated by approximately 375 km. Plants inhabit clay or gravelly silt loam soils in meadows or marshes inundated by slow‐moving water from adjacent creeks, and they occur as rhizomatous clusters of tillers that may form dense patches or occur as more spatially dispersed individuals depending on site conditions. The extent to which neighboring tillers represent the same genet was unknown prior to this study. Plant distribution varied among sites, ranging from large, dense stands containing thousands of apparent plants to smaller and more sparsely distributed occurrences (ORBIC 2024). Unsuccessful surveys for additional locations in similar habitats suggest this species is naturally rare, though land‐use change may have altered its historical distribution (Gisler and Meinke 2001).

Individuals from the North and South naturally occurring sites differ morphologically when grown from seed in a common setting (Gisler and Meinke 2003), suggesting low gene flow and potential adaptive divergence. Some vegetative characteristics, such as plant height, lower leaf size, and ligule length, are significantly greater in plants from the South, but lengths of some floret and spikelet characters are greater in plants from the North. *Pleuropogon oregonus* is gynomonoecious: the uppermost floret is reduced, upper florets are usually pistillate, and lower florets are usually perfect. Anthesis is initially protogynous, initiating with the upper florets, followed by protandrous, starting from the lowest perfect floret upwards (But et al. 1985). Time gaps between anther and style exertion are in minutes to hours (But 1977), facilitating a mixture of outcrossing and self‐pollination. Documented seed set is low (But et al. 1985; Gisler and Meinke 2001) and reproduction may be primarily clonal, but pollen viability (87%) and germinability (85%) are high (But et al. 1985; Gisler and Meinke 2001). Seeds do not show signs of dormancy (Gisler and Meinke 2001), suggesting that the seed bank is transient.

*Pleuropogon oregonus* introductions to sites without known past or contemporary populations started in 2002 and continue to the present. Introduction goals included increasing the species' geographic distribution and the number of individuals growing in protected areas. To facilitate introductions, plants were collected infrequently from natural sites as sod. Sod with rhizomes was divided to encourage tiller growth in a nursery setting. Increases occurred in Corvallis, Oregon, which has milder winters compared to the introduction and natural sites. Dense clusters of tillers resulting from increase were then divided, transplanted in small clusters, and monitored over subsequent years. See Brown et al. (2011), Copeland et al. (2023), and Gisler and Meinke (2001) for a detailed account of propagation protocols. As plants in the North and South are geographically and morphologically distinct, propagules used during introduction efforts were sourced by choosing among those available from the closest naturally occurring sites.

### 2.2 Field Sampling and Data Generation

To facilitate field sampling, we identified all naturally occurring sites of *P. oregonus* by utilizing data requested through Oregon Biodiversity Information Center (ORBIC 2024). We collected leaf tissues at all accessible natural sites and two introduction sites in 2022 (Figure 1). We were unable to obtain permission to access several private properties that contain small populations. In the North, we sampled one natural site (Upper Ladd Canyon) and one introduction site. The vegetative material used for introductions in the North came from two sources. Hereafter, we use I1 to refer to plants grown from rhizomes sourced from a naturally occurring site that we did not sample (Middle Ladd Canyon, approximately 750 m west of Upper Ladd Canyon; ORBIC 2024); these introduction efforts occurred prior to 2012. We use I2 to refer to plants grown from rhizomes sourced from Upper Ladd Canyon and established from 2012 to the present. In the South, we sampled three natural sites (Mud Creek [hereafter Mud], Camas Creek #2 [hereafter Camas], and Horse Prairie [hereafter Horse]) and one introduction site (I3; Figure 1 and Table S1; ORBIC 2024). We collected leaf tissues from 3 to 26 plants per site depending on the number of ramets observed (Table S1). Because the extent and spatial distribution of clonality had not been previously characterized in *P. oregonus* , genetically distinct individuals could not be identified in the field. Rather than imposing a minimum‐distance criterion, we sought to maximize spatial coverage within each site by sampling plants distributed throughout the occupied area whenever possible. At sites containing multiple apparent patches of plants, sampling was distributed among patches to improve representation of site‐wide diversity. Leaf tissues were stored in silica desiccant prior to DNA extraction. In addition, we obtained leaf tissues from ten *P. refractus* individuals to represent an outgroup in genomic analyses. DNA was extracted using DNeasy 96 Plant Kits (Qiagen, Germantown, MD, USA).

We individually barcoded and processed genomic DNAs from 138 individuals into libraries using a restriction fragment‐based procedure (Peterson et al. 2012) modified to utilize separate indexing reads. Samples were randomly assigned to positions in 96‐well plates prior to library preparation. Briefly, we digested DNA using EcoRI and MspI restriction enzymes and ligated Illumina adaptor sequences and barcodes. We barcoded each individual using a unique combination of forward and reverse indices and pooled ligation products, which we amplified using 18 cycles of PCR. We used a Pippin Prep (Sage Science, Beverly, MA, USA) to size select amplicons from 400 to 600 base pairs and sent one library to be sequenced on a NovaSeq 6000 (Illumina, San Diego, CA, USA) at the University of Oregon's Genomics and Cell Characterization Core Facility to generate single‐end 118 base pair reads.

### 2.3 Data Processing, SNP Calling, and Summary Statistics

We demultiplexed and cleaned raw data to exclude raw reads containing more than four low‐quality sites and/or adapter contamination using *fastq‐multx* in ea‐utils (Aronesty 2011) and *process_radtags* in stacks v2.65 (Catchen et al. 2013). We accomplished the remaining processing using stacks and followed the r80 protocol detailed in Rochette and Catchen (2017). We assessed parameters affecting the assembly by quantifying how parameter combinations affected r80 loci (i.e., those found in 80% of samples or more); we selected the optimal parameter set associated with the plateau of the number of r80 loci (Paris et al. 2017; Rochette and Catchen 2017). Parameter values used in our final assembly were: minimum depth of coverage to initiate a new stack (−m in ustacks) = 3; number of mismatches allowed between stacks (−M in ustacks) = 3; distance allowed between catalog loci (−n in cstacks) = 3.

We executed the *populations* program in stacks twice to generate the datasets used in our analyses. Our first run allowed all single nucleotide polymorphisms (SNPs) per locus to be considered and resulted in a dataset used to calculate sampling site summary statistics (observed heterozygosity [*H*_*OBS*_], expected heterozygosity [*H*_*EXP*_], nucleotide diversity [*π*], and inbreeding coefficient [*F*_*IS*_]) and pairwise *F*_*ST*_ values between sampling sites. Ploidy has not, to our knowledge, been directly characterized for *P. oregonus* . Because ploidy variation has been documented in at least one congener, *P. hooverianus* , we interpret heterozygosity and inbreeding coefficients cautiously. Our SNP‐calling and filtering approach assumed diploid genotypes, and unresolved polyploidy or ploidy variation could influence estimates of observed heterozygosity, expected heterozygosity, and *F*_*IS*_. Parameters included: minimum minor allele frequency required to process a nucleotide site at a locus (‐‐min_maf) = 0.02, maximum observed heterozygosity required to process a nucleotide site at a locus (‐‐max_obs_het) = 0.7, and the correction applied to *F*_*ST*_ values (‐‐fst_correction) = *p*_value. In our second run, one SNP per locus was randomly chosen to comply with the assumptions of statistical inferences requiring independence across loci. Parameters were the same as in our first run except for ‐‐write‐random‐snp. Furthermore, to ensure missing data across loci did not influence patterns in allele frequencies across individuals and sites, we excluded SNPs with greater than 10% missing data from both datasets. We estimated pairwise relatedness among individuals within sites (both naturally occurring and introduced) and within the North and South using the KING estimator in vcftools (Danecek et al. 2011; Manichaikul et al. 2010) (‐‐relatedness2) and delineated pairwise individual relationships as clonal or 1° relative based on patterns of empirical clustering around the strict method‐defined thresholds (clonal: *r* > 0.354 and 1° relative: 0.177 < *r* < 0.354). For each sampling site, we quantified clonal richness as $R=\left(G-1\right)/\left(N-1\right)$, where G is the number of inferred genets and N is the number of sampled ramets (Dorken and Eckert 2001).

### 2.4 Population Structure and Differentiation

We used two approaches to investigate genomic structure. First, we implemented Bayesian clustering using structure v2.3.4 (Falush et al. 2007) across *K*‐values ranging from 1 to 9 without assigning sampling site membership a priori. We conducted twenty independent runs per *K*, each with 100,000 burn‐in and 250,000 Markov chain Monte Carlo iterations, using an admixture model with correlated allele frequencies. We used structure harvester and distruct to visualize results. Second, we implemented principal component analysis (PCA) using the *adegenet* package (Jombart et al. 2008) in R to complement structure analyses given each method's reliance on different assumptions (Frantz et al. 2008; Jombart et al. 2008). We assessed results of these analyses in tandem, along with pairwise *F*_*ST*_, to determine biologically relevant, genomically defined groups instead of using model selection methods that can be misleading (i.e., Janes et al. 2017). To ensure that population genetic patterns were not biased by clones in our datasets, we reran structure and PCA using only one representative individual per clone. Genomic structure analyses inform whether introduced individuals reflect underlying evolutionary lineages occurring in the natural populations.

### 2.5 Demographic Inference

Coalescent simulations can be applied to robustly estimate site frequency spectra under complex demographic scenarios, which facilitates parameter estimation and model selection based on fit of simulated to empirical data. We developed two models to understand whether historical gene flow occurred between the naturally occurring sites (i.e., populations according to population genetic results, see below) in the North and South, as well as the approximate timing of their divergence. Specifically, we tested strict isolation (SI) and isolation‐with‐migration (IM) models using fastsimcoal2 v2.8 (Excoffier et al. 2021; Figure S1). Support for the SI model would indicate that gene flow has not occurred between North and South populations since divergence, while support for the IM model would indicate post‐divergence gene flow. For fastsimcoal2 simulations, we only included individuals from naturally occurring locations and one individual per clone. We estimated the composite likelihood of the observed data given a specified model using the folded joint site frequency spectrum (SFS), which we calculated from the dataset including one single nucleotide polymorphism (SNP) per locus (see above) to avoid the effects of linkage disequilibrium.

As we did not include invariable sites in the SFS, we fixed the effective population size of the North population to enable the estimation of the other parameters in fastsimcoal2 (Excoffier et al. 2013). We estimated the contemporary effective population size (*Ne*) using the *ldNe* function from the *StrataG* R package (Archer et al. 2017) per methods described by Waples et al. (2016). To remove all missing data for calculation of the joint SFS, minimize errors in allele frequency estimates, and maximize the number of variable SNPs retained, we projected *Pleuropogon* populations down to 8 and 3 individuals from the North and South populations, respectively; sample sizes were limited because we only included one individual per clone. We constructed projections using the *easySFS.py* script (I. Overcast, https://github.com/isaacovercast/easySFS). We ran each model 100 times using 1,000,000 simulations for the calculation of composite likelihood, 100 expectation‐conditional maximization (ECM) cycles, and a stopping criterion of 0.0001. In addition, we constrained the number of unsuccessful ECM cycles to 5 before resetting parameters to those prevailing after the last maximum likelihood improvement.

We used an information‐theoretic model selection approach based on Akaike's information criterion (AIC) to compare the models and identify the one that was best supported by the data. After the maximum likelihood was estimated for both models in every replicate, we calculated AIC scores as detailed in Anderson and Burnham (2002). We selected point estimates of the demographic parameters from the run with the highest maximum composite likelihood. Finally, we calculated confidence intervals (based on the percentile method) of parameter estimates from 100 parametric bootstrap replicates by simulating the SFS from the maximum composite likelihood estimates and re‐estimating parameters each time (Excoffier et al. 2013). While interpreting absolute divergence times based on generations is difficult, field‐based observations suggest that *Pleuropogon* may become reproductive in 1 year (on average). These analyses provide evolutionary context for evaluating whether historical isolation constrains contemporary management actions such as regional mixing and whether distinct lineages may best be managed independently.

## 3 Results

### 3.1 Next‐Generation Sequencing Data and Genetic Summary Statistics

Illumina sequencing produced > 3.4 × 10^8^ reads across 138 individuals (average of 2.48 × 10^6^ reads per individual). Two individuals were removed due to low coverage of usable, processed reads (Table S1). After removing low‐quality reads and adapter contamination, > 2.9 × 10^8^ reads remained to identify homologous loci and SNPs in stacks (Table S1). The dataset including all SNPs per locus was used to calculate sampling site summary statistics and pairwise *F*_*ST*_ values and contained 364,245 loci and 60,342 variant sites. Effective per‐sample coverage had a mean of 52.8×. After selecting one SNP per locus and filtering loci with > 10% missing data, 7649 unlinked SNPs remained for PCA and structure analyses. Heterozygosity (*H*_*OBS*_, *H*_*EXP*_) and nucleotide diversity (*π*) were consistent within the North and South *P. oregonus* sampling sites, regardless of whether the site was natural or introduced, and the inbreeding coefficient (*F*_*IS*_) was uniformly low (Table 1). Notably, both heterozygosity and nucleotide diversity were higher in North sampling sites compared to South sampling sites. *F*_*ST*_ values between North and South naturally occurring *P. oregonus* sites ranged from 0.21 to 0.31, while *F*_*ST*_ between natural and introduced sites within regions was lower (Upper Ladd Canyon versus I1/I2: 0.02–0.09; Camas/Horse/Mud versus I3: 0.05–0.17; Table 2). Average *F*_*ST*_ between *P. oregonus* sampling sites and *P. refractus* was 0.76, indicating substantial genetic differentiation. These patterns indicate that individuals at introduced sites generally reflect the genetic diversity present within their source regions, while strong divergence between regions suggests that genetic variation is structured at broader spatial scales relevant to sourcing decisions.

**TABLE 1:** Genomic summary statistics calculated using variant sites for *Pleuropogon* sampling sources, including private alleles (Private); average sample size used to calculate statistics across loci (#Indv ± SE); observed heterozygosity (*H*_*OBS*_ ± SE); expected heterozygosity (*H*_*EXP*_ ± SE); nucleotide diversity (*π* ± SE); and inbreeding coefficient (*F*_*IS*_ ± SE).

Sampling site	Population	Private	#Indv ± SE	*H*_*OBS*_ ± SE	*H*_*EXP*_ ± SE	*π* ± SE	*F*_*IS*_ ± SE
Upper Ladd Canyon	North	1339	15.35 ± 0.04	0.212 ± 0.001	0.219 ± 0.001	0.226 ± 0.001	0.03 ± 0.04
I1	North	3403	14.64 ± 0.03	0.195 ± 0.001	0.199 ± 0.001	0.213 ± 0.001	0.05 ± 0.03
I2	North	1005	15.52 ± 0.02	0.224 ± 0.002	0.179 ± 0.001	0.211 ± 0.001	−0.01 ± 0.01
Camas	South	1272	16.86 ± 0.04	0.127 ± 0.001	0.096 ± 0.001	0.105 ± 0.001	−0.04 ± 0.04
Horse	South	2223	11.58 ± 0.03	0.146 ± 0.001	0.105 ± 0.001	0.118 ± 0.001	−0.05 ± 0.03
Mud	South	673	2.35 ± 0.00	0.129 ± 0.002	0.077 ± 0.001	0.108 ± 0.001	−0.03 ± 0.00
I3	South	805	5.58 ± 0.01	0.159 ± 0.002	0.101 ± 0.001	0.123 ± 0.001	−0.07 ± 0.01
*P. refractus*	n/a	2156	6.31 ± 0.01	0.221 ± 0.001	0.202 ± 0.001	0.232 ± 0.001	0.03 ± 0.02

**TABLE 2:** Pairwise *F*_*ST*_ values between *P. oregonus* sampling sites and with *P. refractus* (outgroup taxon). ULC = Upper Ladd Canyon. *F*_*ST*_ values ranged from 0.02 to 0.10 in North–North site comparisons, 0.05–0.17 in South–South site comparisons, and 0.21–0.38 in North–South site comparisons.

	I1	I2	Camas	Horse	Mud	I3	*P. refractus*
ULC	0.09	0.02	0.31	0.29	0.21	0.25	0.72
I1		0.10	0.33	0.31	0.24	0.27	0.73
I2			0.38	0.35	0.27	0.33	0.72
Camas				0.17	0.12	0.10	0.78
Horse					0.15	0.16	0.79
Mud						0.05	0.80
I3							0.80

### 3.2 Relatedness Among Individuals Within and Between Sites

**FIGURE 2:**
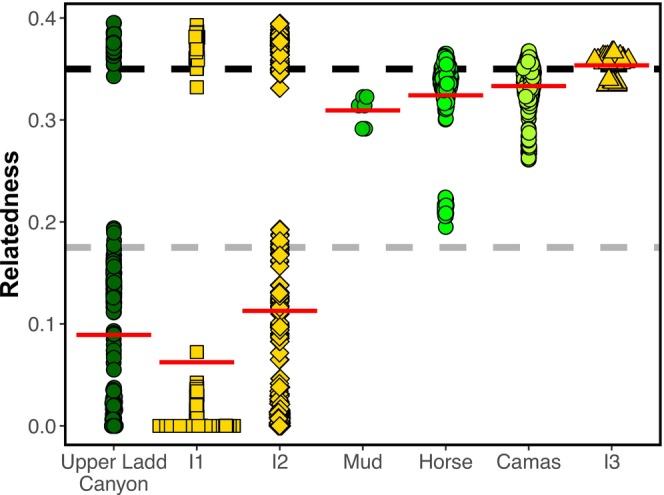
Relatedness (estimated kinship coefficients using the KING estimator; Manichaikul et al. 2010) within sites. Points represent kinship coefficients for within‐site pairs of individuals. Red bars are mean within‐site coefficient values. The black dashed line indicates the threshold value for clonal relationships (r > 0.354). The grey dashed line indicates the threshold for 1° relationships (e.g., parent‐offspring or full‐sibling pairs). Values of 0 indicate unrelated pairs (Manichaikul et al. 2010). Green circles represent naturally occurring sites, while yellow shapes indicate plants established at Introduction sites. Colors and shapes of symbols representing sampling sites are consistent with Figures 1 and 3.

Relationships between individuals within sites varied from clonal to unrelated. In the North, the natural site (Upper Ladd Canyon) contained a diverse suite of relationships and sampled individuals belonged to one of eight clones (Figure 2). The introduction sites (I1 and I2), which were established using plants grown from field‐harvested rhizomes prior to knowing relatedness estimated from genetic data, contained both clones and unrelated individuals (Figure 2). Between‐site comparisons revealed that individuals from Upper Ladd Canyon were all unrelated to individuals sampled at I1, while a range of related and unrelated relationships were identified between Upper Ladd Canyon and I2 (Figure S2). In contrast to Upper Ladd Canyon, we identified all individuals sampled within each naturally occurring South site (Camas, Horse, and Mud) as clones or 1° relatives (Figure 2). Similarly, all individuals sampled from the South introduction site (I3) were clones, though clonal relationships were not identified between individuals at I3 and any of the naturally occurring sites (Figure S3). We acknowledge that clone identification was based on the empirical clustering of pairwise relationships around the theoretical relatedness thresholds derived from Manichaikul et al. (2010) rather than on calibrated thresholds derived from known replicate samples. Consequently, relationships between individuals should be interpreted cautiously and are best viewed as suggestive of clonal and degrees of non‐clonal relatedness. Additional empirical validation would provide greater confidence in clone delineation. Patterns of relatedness within and between sites suggest that the effectiveness of vegetative propagation in capturing genetic diversity depends strongly on the diversity present at the sources. Although smaller sample sizes from the South sites may increase uncertainty, they contained fewer genetically distinct individuals compared to Upper Ladd Canyon, indicating higher diversity among sampled plants in the North and more limited sampled diversity in the South.

### 3.3 Population Structure and History

**FIGURE 3:**
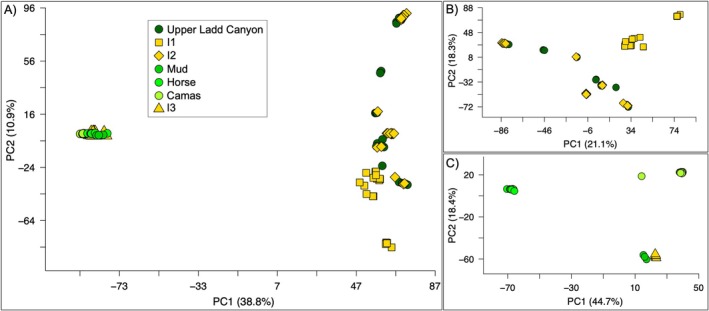
Principal component analyses for A) all sampled *P. oregonus* individuals, B) North population sampling sites, and C) South population sampling sites. Colors and shapes are consistent throughout panels A, B, and C, with introduction sites represented by yellow shapes and naturally occurring sites represented by green circles. Colors and shapes of symbols are consistent with Figures 1 and 2. Variation explained by principal component (PC) axes are provided in parentheses.

Population structure analyses provided consistent support for two genomic groups corresponding to the North and South regions we define using geography (Figure 1). Hereafter, we refer to all individuals occurring or originating from the naturally occurring North sites as the North population and all individuals occurring or originating from the naturally occurring South sites as the South population. PCA on all individuals differentiated the North and South populations on PC1, while variation within the North population drove patterns among individuals on PC2 (Figure 3A). Clusters of individuals from Upper Ladd Canyon mirror clonal identity quantified in relatedness analyses (Figure 2); in other words, there are eight clusters of individuals each representing one clone, and three of the clones are closely related (and thus cluster closely in PC space). Within the North population, introduced individuals from I2 overlap substantially with individuals from Upper Ladd Canyon, while individuals from I1 occupy unique PC space (Figure 3B). Individuals show high fidelity to sampling sites in PCA focused on South population individuals, and individuals from the I3 introduction site cluster tightly with individuals from Mud (Figure 3C). Principal components results were reiterated in structure analyses, wherein the primary axis of genomic differentiation (i.e., *K* = 2) was between the North and South populations (Figure S4). *K* = 3 identifies variation within the North population that is not consistent with a sampling site, while individuals from the I1 introduction site that occupy unique PC space in Figure 3B are identified at *K* = 4. Higher genomic axes (*K* = 5 and above) split variation within the South population, similar to PCA patterns (Figure 3C). Rarefaction analyses using only non‐clonal individuals did not change patterns reported when considering all individuals (Figures S5 and S6). Population structure results indicate that introduced individuals largely preserve regional genetic structure, while the strong and consistent divergence between North and South populations suggests that these regions represent distinct evolutionary lineages relevant to management decisions about population mixing.

### 3.4 Demographic Model Selection and Parameter Estimation

Coalescent modeling using fastsimcoal2 resulted in overwhelming support for the isolation‐with‐migration (IM) model compared to a strict isolation (SI) model (∆AIC = 15,520; Table 3). Estimates of contemporary effective population size were low for both *P. oregonus* populations, a pattern expected in highly clonal species where relatively few genetically distinct individuals contribute to the gene pool, even when census sizes are larger. In this system, effective population size reflects the number of unique genets rather than the abundance of ramets and therefore cannot be interpreted as evidence of demographic collapse or poor population performance. Under the IM model, the North and South populations diverged approximately 160,000 generations before present; assuming *P. oregonus* becomes reproductive 1 year after germination and establishment, this result suggests that population divergence commenced prior to the start of the last glacial period (Clark et al. 2009). Ancestral population size (θ_*ANC*_) was estimated to be large (~127,000 individuals), while ongoing gene flow after divergence was estimated to be low (0.02–0.03). These models are simplified representations of *P. oregonus* population divergence; we tested between them herein only to understand the general mode and tempo influencing our focal species. In addition, parameter estimates themselves are best treated as heuristic, not as quantitative reconstructions. These results indicate long‐term evolutionary separation between regions with limited historical connectivity, providing context for evaluating future sourcing and mixing decisions.

**TABLE 3:** Parameters estimated using coalescent simulations with fastsimcoal2 for each model of divergence between the North and South populations (illustrated in Figure S1). The table shows point estimates and lower and upper 95% confidence intervals for each parameter, which include ancestral (θ_*ANC*_ *)* and contemporary effective population size (θ_*2*_), effective migration rates per generation (*m*_*12*_ and *m*_*21*_), and timing of population divergence (*T*_*DIV*_). Estimates are given in years, considering generation time of 1 year. Effective population size of one population (θ_*1*_) was calculated using the *ldNe* function from the *StrataG* R package (Archer et al. 2017) per methods described by Waples et al. (2016) and fixed in fastsimcoal2 analyses to enable the estimation of other parameters. lnL is the maximum likelihood value of the model; *k* is the number of parameters in the model; ∆AIC is the difference in AIC values from the strongest model.

Model	θ_*ANC*_	θ_*2*_	*m*_*12*_	*m*_*21*_	*T*_*DIV*_	lnL	*k*	AIC	∆AIC
Isolation with Migration	127,167 ± 33,902	3 ± 2	0.03 ± 0.02	0.02 ± 0.01	160,136 ± 59,618	−11786.7	5	23,583	0
Strict Isolation	318 ± 79	65 ± 13			100 ± 42	−19548.9	3	39,104	15,520

## 4 Discussion

Conservation translocations are widely used to increase population size and reduce extinction risk, yet their genetic consequences remain uncertain, particularly for species propagated vegetatively. Here, we evaluated whether vegetative propagation preserves genomic diversity and informs sourcing decisions using a rare, clonal plant system. Our results demonstrate that vegetative propagation can retain genetic diversity and capture multiple distinct genotypes, thereby increasing population redundancy, but that its effectiveness depends strongly on the diversity present in source populations. At the same time, deep evolutionary divergence and limited gene flow between regions indicate that mixing populations may risk disrupting independently evolving lineages. Together, these findings highlight that vegetative propagation can be an effective conservation strategy when applied within an evolutionary framework that accounts for population structure and divergence history.

### 4.1 *Pleuropogon oregonus* Is Composed of Genetically Divergent Populations With High Clonality

The contemporary distribution of *P. oregonus* is limited to a few naturally occurring sites separated by 375 km, and genomic patterns among individuals composing the disjunct North and South populations reflect independent evolutionary trajectories of these populations. All analyses, including *F*_*ST*_, structure, and PCA, indicate that the primary axis of differentiation is between North and South, rather than among sites or individuals within these regions (Figure 3 and Table 2). The North population, represented in our dataset by one naturally occurring site (Upper Ladd Canyon), contains at least eight genetically distinct clones that grow amongst one another. In addition, genetic analyses of individuals sampled from the I1 introduction, which was documented to have used propagules from Middle Ladd Canyon, a distinct natural site approximately 750 m from Upper Ladd Canyon, suggested that additional genetic variation may be present on privately owned land. In contrast, the South population consists of naturally occurring sites that are each dominated by one clone or a mix of clones and 1° relatives. Within the South population, sampled individuals from different sites were generally not inferred to be clonal or close relatives, suggesting that each site contains distinct genets or groups of related individuals. However, differentiation among South sites was low relative to the divergence between the North and South populations, and structure provided little evidence for discrete South populations. Thus, genetic patterns within the South are best interpreted as weak site‐level structure among distinct local genets rather than evidence for complete isolation among sites.

Considered together, our results indicate that there are few genetically distinct individuals across the range of *P. oregonus* , though we acknowledge that we did not thoroughly sample sites containing thousands of ramets (e.g., Upper Ladd Canyon and Camas), where sampling was intended to capture site‐wide genetic diversity rather than exhaustively characterize all genets. However, populations with limited numbers of genetically distinct individuals are not necessarily biologically unsuccessful. For example, clones of *Andryala laevitomentosa*, represented by 11 genetic individuals across the species' extant distribution, are estimated to be 24–64 thousand years old (Mráz et al. 2025). Under the assumption that clones may persist for millennia, habitat protection from anthropogenic factors such as habitat disturbance or destruction is critical (Thuiller et al. 2008; Lavergne et al. 2010). We reiterate that effective population size estimates are most informative here in a comparative and management context, highlighting differences in genetic contribution among populations rather than providing absolute measures of viability. We also acknowledge that clone identification was based on theoretical relatedness thresholds rather than empirically calibrated thresholds derived from known replicate samples. While the exact number of inferred clones may therefore vary depending on the threshold applied, major conclusions regarding regional divergence, population structure, and the genetic composition of introduced populations were supported by multiple independent analyses and are therefore robust to uncertainty in clone delineation.

Clonal wetland species display wide variation in genetic diversity, heterozygosity, inbreeding, and genetic structure, making comparisons among species useful but context‐dependent. Within our study, inbreeding coefficients were uniformly low, but the North population contained higher genetic diversity and heterozygosity compared to the South population (Table 1). Ultimately, this pattern may result from the North population containing more genetically unique individuals compared to the South population, which may be losing clones through genetic drift or similar processes (Chung et al. 2004). Low inbreeding coefficients in *P. oregonus* may also reflect the persistence of heterozygous genotypes through clonal reproduction and limited opportunity for repeated sexual reproduction among close relatives. In addition, ploidy has not, to our knowledge, been directly characterized in *P. oregonus* , and ploidy variation has been documented in at least one congener, *P. hooverianus* . As such, unresolved ploidy variation could influence estimates of observed heterozygosity, expected heterozygosity, and *F*_*IS*_, and these summary statistics should be interpreted cautiously. Regardless, the high differentiation observed in our samples does not seem to simply reflect a pattern of low genetic diversity (see Jakobsson et al. 2013), and our major conclusions rely primarily on concordant patterns of population structure, differentiation, relatedness, and introduction‐source affinity rather than absolute values of *F*_*IS*_ alone. Across other wetland species, the rare clonal grass *Xyris tennesseensis* displayed observed heterozygosity values much lower than *P. oregonus* (Downey and Baskauf 2020). Meanwhile, the rare clonal wetland species *Lilaeopsis shaffneriana* subsp. *recurva* had substantially higher observed heterozygosity and little indication of inbreeding (Fehlberg 2017). In two common *Carex* species, *C. lasiocarpa* and *C. pellita* , observed heterozygosity roughly spanned a range comparable to the two *P. oregonus* populations (McClintock and Waterway 1993). As such, comparison of genetic diversity values between *P. oregonus* and other species provides little indication of whether observed values may be biologically relevant.

Many plants confined to wetland or aquatic habitats are widely distributed (Santamaría 2002) and often display low differentiation across large spatial scales (Sweetman et al. 2013; Kettenring et al. 2019; Lobato‐de Magalhaes et al. 2020). On an ecological timescale, dispersal is a primary vector of gene flow among wetland species' populations, for example as birds carry seeds either on their feet or in their digestive tracts (Mueller and van der Valk 2002; Santamaría 2002). Given that the *P. oregonus* sites are located on the Pacific Flyway (Newcombe et al. 2019), genetic patterns may suggest that *P. oregonus* seeds are not palatable to avian vectors or that *P. oregonus* is so rare that, by chance, propagule pressure is not high enough to effectively promote gene flow between regions. Differentiation may better be explained by processes over an evolutionary timescale, as the divergence history of the North and South *P. oregonus* populations corresponds with timescales associated with glacial–interglacial climatic fluctuations (Table 3). Isolation into geographically distinct refugia was a common mechanism that promoted differentiation across taxa in western North America (Shafer et al. 2010), especially in topographically heterogeneous regions similar to eastern Oregon (Hodel et al. 2021). Furthermore, post‐divergence gene flow between the North and South populations indicated by the isolation‐with‐migration model (Table 3) may have occurred during moister periods when population sizes were larger and distributed across more suitable habitat. In Oregon, there have been several periods since the Last Glacial Maximum that may have favored larger *P. oregonus* populations (Grigg and Whitlock 1998), which would have increased the probability for effective gene flow.

### 4.2 Introduction Using Vegetative Propagules Has Resulted in Genetically Representative Populations

Using rhizomes to produce individuals for planting at introduction sites was conducted without genetic information. Regardless, the long‐term introduction methodology described by Gisler and Meinke (2001), Gisler and Meinke (2003), and Copeland et al. (2023) captured most of the genetically unique individuals that we sampled at the naturally occurring North site (Upper Ladd Canyon; Figure 3). Because the rhizomes were representative of adult plants, differentiation between I2 and Upper Ladd Canyon was low (low pairwise *F*_*ST*_; Table 2) and diversity values were comparable (Table 1). Rhizomes used to produce plants for the I1 introduction were sourced from a natural population located on private land not sampled for genetic analysis herein (Middle Ladd Canyon). Our ability to quantify genetic differences confirms that additional genetically unique individuals may be present on the landscape. However, if the unsampled natural site is extirpated, the I1 introduction site may be the only location containing these unique *P. oregonus* alleles (e.g., I1 has 3403 private alleles; Table 1), which highlights the importance of redundancy for rare species. In the South, genetic analyses suggest that the naturally occurring Mud site may have produced the introduction material, as I3 individuals are minimally different than Mud individuals in PC space (Figure 3C) and have a low *F*_*ST*_ value (Table 2). Notably, if the clones at the other naturally occurring South sites (Camas and Horse; Figure 1) have not been used for introductions, these genotypes have no redundancy across the landscape and could be targeted for collection for future introduction efforts. While vegetative propagules have the capacity to create genetically redundant populations even in the absence of genetic information, genetic study prior to the production of plant materials can improve genetic representation (Maschinski and Albrecht 2017). If genetic study is not feasible, sampling efforts would best proceed by including as much of the plants' distribution as possible.

### 4.3 Translocations Benefit From Knowledge About Reproduction

For *P. oregonus* and similarly rare, clonal species, investigating and characterizing the nature of reproduction (sexual versus clonal) and spatial pattern of clones within populations is critical to inform the collection of propagules for translocation. High survivorship of vegetative propagules at the introduction sites reduces the potential for loss of genetic diversity, but using vegetative propagules does not promote new and potentially beneficial genotypes that result from recombination during outcrossing, which may be beneficial for a species to navigate changing environmental conditions (Barton and Charlesworth 1998). Moreover, the nature of reproduction within populations may provide insight into functional connectivity, pollen and seed dispersal, heterozygosity, and inbreeding. While *P. oregonus* seeds have been documented to have high germinability, ranging from 48% to 90% (But et al. 1985; Gisler and Meinke 2001; Copeland et al. 2023), But et al. (1985) noted relatively low seed set and suggest *P. oregonus* may not be self‐compatible. As such, low clonal diversity at naturally occurring *P. oregonus* sites may pose barriers to outcrossing. For example, unsuccessful sexual reproduction was documented in the rare, clonal twinflower ( *Linnaea borealis* ) and attributed to a high rate of within‐clone pollination (Wilcock 2002). Ensuring clonal diversity at introduction sites may therefore be best practice, and admixture could increase genetic diversity and adaptive potential where populations have low effective population sizes or few distinct genets. However, the length of isolation between the *P. oregonus* North and South populations suggests that interregional mixing must be approached cautiously, particularly when considering augmentation of naturally occurring sites (Frankham et al. 2011). Because our study did not directly evaluate local adaptation, outbreeding depression, or the fitness consequences of admixture, the relative benefits and risks of mixing North and South lineages remain uncertain.

### 4.4 Implications for Conservation Practice

Our results provide several general insights for conservation translocations involving clonal species. First, vegetative propagation can effectively preserve genomic diversity when propagules are collected from multiple genetically distinct individuals. Second, sampling strategies that prioritize spatial breadth within populations, particularly in clonal systems where many ramets may represent the same genet, may maximize the probability of capturing distinct genotypes. Third, strong and persistent genetic divergence among populations suggests that mixing material across regions must be approached cautiously, as such populations may represent independently evolving lineages with limited historical gene flow. Fourth, introduction efforts can increase population redundancy and safeguard genetic variation, including alleles from unsampled or extirpated populations. Fifth, genomic data collected prior to and following translocation can substantially improve the design and evaluation of conservation interventions by revealing patterns of diversity, relatedness, and lineage structure that are not detectable using field observation, census counts, morphology, or source‐location records alone (Van Rossum and Le Pajolec 2021). Finally, we reiterate that seed‐based introductions and introductions using vegetative propagules may differ in how genetic diversity is transmitted from source material to established populations. Seed‐based introductions may generate new recombinant genotypes, but the genetic composition of established plants can be shaped by seed production, germination, establishment, and early survival filters. In contrast, vegetative propagation can more directly preserve sampled genotypes because propagated ramets bypass seed‐germination filters and can retain genetic variation present in collected source material. However, both approaches are limited by the diversity present in source populations and by the extent to which propagules are sampled representatively.

## Funding

This project was funded by the U.S. Fish and Wildlife Service Tribal Grant 21AP00710, U.S. Geological Survey Southwest Biological Science Center, and Landscape Stewardship Collective.

## Conflicts of Interest

The authors declare no conflicts of interest.

## Supporting information


**Table S1:** Sampling locations, clonal diversity metrics, and data‐processing details. Columns indicate the number of sampled ramets (N), inferred genets (G), and clonal richness (R = [G − 1]/[*N* − 1]) for each sampling locality. Values in parentheses indicate the number of individuals retained after data‐processing filters were applied. Processed reads indicate the number of reads per individual used for locus construction (ustacks; stacks). Sampling localities are categorized as naturally occurring populations or introduction sites. *Pleuropogon refractus* represents the outgroup taxon.
**Figure S1:** Divergence models tested using fastsimcoal2, including strict isolation (SI) and isolation‐with‐migration (IM). Parameters include: mutation‐scaled ancestral (θ_*ANC*_); contemporary effective population sizes (θ_*1*_ and θ_*2*_); effective migration rates per generation (*m*_*12*_ and *m*_*21*_); and timing of population divergence (*T*_*DIV*_).
**Figure S2:** Estimated kinship coefficients calculated using individuals from different sampling sites in the North population using the KING estimator for relatedness. Points represent kinship coefficients for between‐site sample pairs. Points represent kinship coefficients from between‐sample comparisons. Red bars are mean within‐site coefficient values. The black dashed line indicates the threshold value for clonal relationships (*r* > 0.354). The gray dashed line indicates the threshold for 1° relationships (e.g., parent‐offspring or full‐sibling pairs). Values of 0 indicate unrelated pairs (Manichaikul et al. 2010).
**Figure S3:** Estimated kinship coefficients calculated using individuals from different sampling sites in the South population using the KING estimator for relatedness. Points represent kinship coefficients for between‐site sample pairs. Red bars are mean within‐site coefficient values. The black dashed line indicates the threshold value for clonal relationships (*r* > 0.354). The gray dashed line indicates the threshold for 1° relationships (e.g., parent‐offspring or full‐sibling pairs). Values of 0 indicate unrelated pairs (Manichaikul et al. 2010).
**Figure S4:** structure results for analyses of *K*‐values 2–5. Population designations are noted along the top of *K* = 2 and sites names (as displayed in Figure 1) are shown across the bottom of *K* = 5. Solid vertical black lines delineate sampling sites. ULC = Upper Ladd Canyon.
**Figure S5:** Principal component analyses including only non‐clonal *P. oregonus* individuals. Colors and shapes are consistent with Figure 3. Variation explained by PC axes are provided in parentheses.
**Figure S6:** structure results for analyses of *K*‐values 2–5 using only individuals identified as non‐clones. Population designations are noted along the top of *K* = 2 and sites names (as displayed in Figure 1) are shown across the bottom of *K* = 5. Solid vertical black lines delineate sampling sites. ULC = Upper Ladd Canyon.

## Data Availability

Data for this study are available at Dryad: doi:10.5061/dryad.qz612jmxm.
