# Supplementary material for: Vegetative Propagation Preserves Genomic Diversity and Informs Translocation Strategies in a Rare Clonal Plant
Source: Evol Appl. 2026 Jul 18;19(7):e70299. doi: 10.1111/eva.70299 (PMC13379775; doi:10.1111/eva.70299)
Supplement: Supplementary file 1 — Table S1: Sampling locations, clonal diversity metrics, and data‐processing details. Columns indicate the number of sampled ramets (N), inferred genets (G), and clonal richness (R = [G − 1]/[N − 1]) for each sampling locality. Values in parentheses indicate the number of individuals retained after data‐processing filters were applied. Processed reads indicate the number of reads per individual used for locus construction (ustacks; stacks). Sampling localities are categorized as naturally occurring populations or introduction sites. Pleuropogon refractus represents the outgroup taxon. Figure S1: Divergence models tested using fastsimcoal2, including strict isolation (SI) and isolation‐with‐migration (IM). Parameters include: mutation‐scaled ancestral (θ ANC ); contemporary effective population sizes (θ 1 and θ 2 ); effective migration rates per generation (m 12 and m 21 ); and timing of population divergence (T DIV ). Figure S2: Estimated kinship coefficients calculated using individuals from different sampling sites in the North population using the KING estimator for relatedness. Points represent kinship coefficients for between‐site sample pairs. Points represent kinship coefficients from between‐sample comparisons. Red bars are mean within‐site coefficient values. The black dashed line indicates the threshold value for clonal relationships (r > 0.354). The gray dashed line indicates the threshold for 1° relationships (e.g., parent‐offspring or full‐sibling pairs). Values of 0 indicate unrelated pairs (Manichaikul et al. 2010). Figure S3: Estimated kinship coefficients calculated using individuals from different sampling sites in the South population using the KING estimator for relatedness. Points represent kinship coefficients for between‐site sample pairs. Red bars are mean within‐site coefficient values. The black dashed line indicates the threshold value for clonal relationships (r > 0.354). The gray dashed line indicates the threshold for 1° relationships (e [file EVA-19-e70299-s001.docx]

Supplemental Information for: Vegetative propagation preserves genomic diversity and informs translocation strategies in a rare clonal plant

Rob Massatti^1*^, Susan Bainbridge^2^, Stella M. Copeland^3^, Carter Crouch^4^, Trevor M. Faske^1^, Erik Hamerlynck^3^, Brandon Palmer^5^, Carla Roybal^2^

^1^Landscape Stewardship Collective, Flagstaff, AZ 86004
^2^U.S. Geological Survey Southwest Biological Science Center, Flagstaff, AZ 86004

^3^USDA-Agricultural Research Service, Eastern Oregon Agricultural Research Center, Burns, OR 97720

^4^International Crane Foundation, Rockport, TX, USA

^5^Oregon Desert Land Trust, Burns, OR, USA

*Corresponding author: [rob@landscollective.org](mailto:rob@landscollective.org)

**Table S1.** Sampling locations, clonal diversity metrics, and data-processing details. Columns indicate the number of sampled ramets (N), inferred genets (G), and clonal richness (R = [G−1]/[N−1]) for each sampling locality. Values in parentheses indicate the number of individuals retained after data-processing filters were applied. Processed reads indicate the number of reads per individual used for locus construction (ustacks; stacks). Sampling localities are categorized as naturally occurring populations or introduction sites. Pleuropogon refractus represents the outgroup taxon.

| **Sampling locality** | **Sampled Ramets (N)** | **Inferred Genets (G)** | **Clonal Richness (R)** | **Population** | **N/R** | **Processed reads (± SD)** | **Utilized reads (± SD)** |
| --- | --- | --- | --- | --- | --- | --- | --- |
| Upper Ladd Canyon | 26 | 8 | 0.28 | North | natural | 2,015,890 ± 291,524 | 1,530,757 ± 231,693 |
| I1 | 23 | 8 | 0.32 | North | introduction | 2,234,720 ± 456,340 | 1,809,372 ± 339,042 |
| I2 | 26 (25) | 7 | 0.24 | North | introduction | 2,315,801 ± 405,093 | 1,799,201 ± 337,539 |
| Camas | 24 | 1 | 0.00 | South | natural | 2,441,496 ± 409,907 | 1,949,873 ± 317,863 |
| Horse | 18 (17) | 2 | 0.06 | South | natural | 2,359,065 ± 357,673 | 1,746,330 ± 471,675 |
| Mud | 3 | 1 | 0.00 | South | natural | 2,394,859 ± 1,198,721 | 1,740822 ± 837,541 |
| I3 | 8 | 1 | 0.00 | South | introduction | 1,995,992 ± 272,877 | 1,437,483 ± 200,485 |
| *P. refractus* | 10 | 10 | 1.00 | n/a | n/a | 1,536,561 ± 547,837 | 843,910 ± 306,011 |

**Figure S1**. Divergence models tested using fastsimcoal2, including strict isolation (SI) and isolation-with-migration (IM). Parameters include: mutation-scaled ancestral ($\theta$*_ANC_*); contemporary effective population sizes ($\theta$*_1_* and $\theta$*_2_*); effective migration rates per generation (*m_12_* and *m_21_*); and timing of population divergence (*T_DIV_*).

**Figure S2**. Estimated kinship coefficients calculated using individuals from different sampling sites in the North population using the KING estimator for relatedness. Points represent kinship coefficients for between-site sample pairs. Points represent kinship coefficients from between-sample comparisons. Red bars are mean within-site coefficient values. The black dashed line indicates the threshold value for clonal relationships (*r* > 0.354). The grey dashed line indicates the threshold for 1° relationships (e.g., parent-offspring or full-sibling pairs). Values of 0 indicate unrelated pairs (Manichaikul et al., 2010).

**Figure S3**. Estimated kinship coefficients calculated using individuals from different sampling sites in the South population using the KING estimator for relatedness. Points represent kinship coefficients for between-site sample pairs. Red bars are mean within-site coefficient values. The black dashed line indicates the threshold value for clonal relationships (*r* > 0.354). The grey dashed line indicates the threshold for 1° relationships (e.g., parent-offspring or full-sibling pairs). Values of 0 indicate unrelated pairs (Manichaikul et al., 2010).


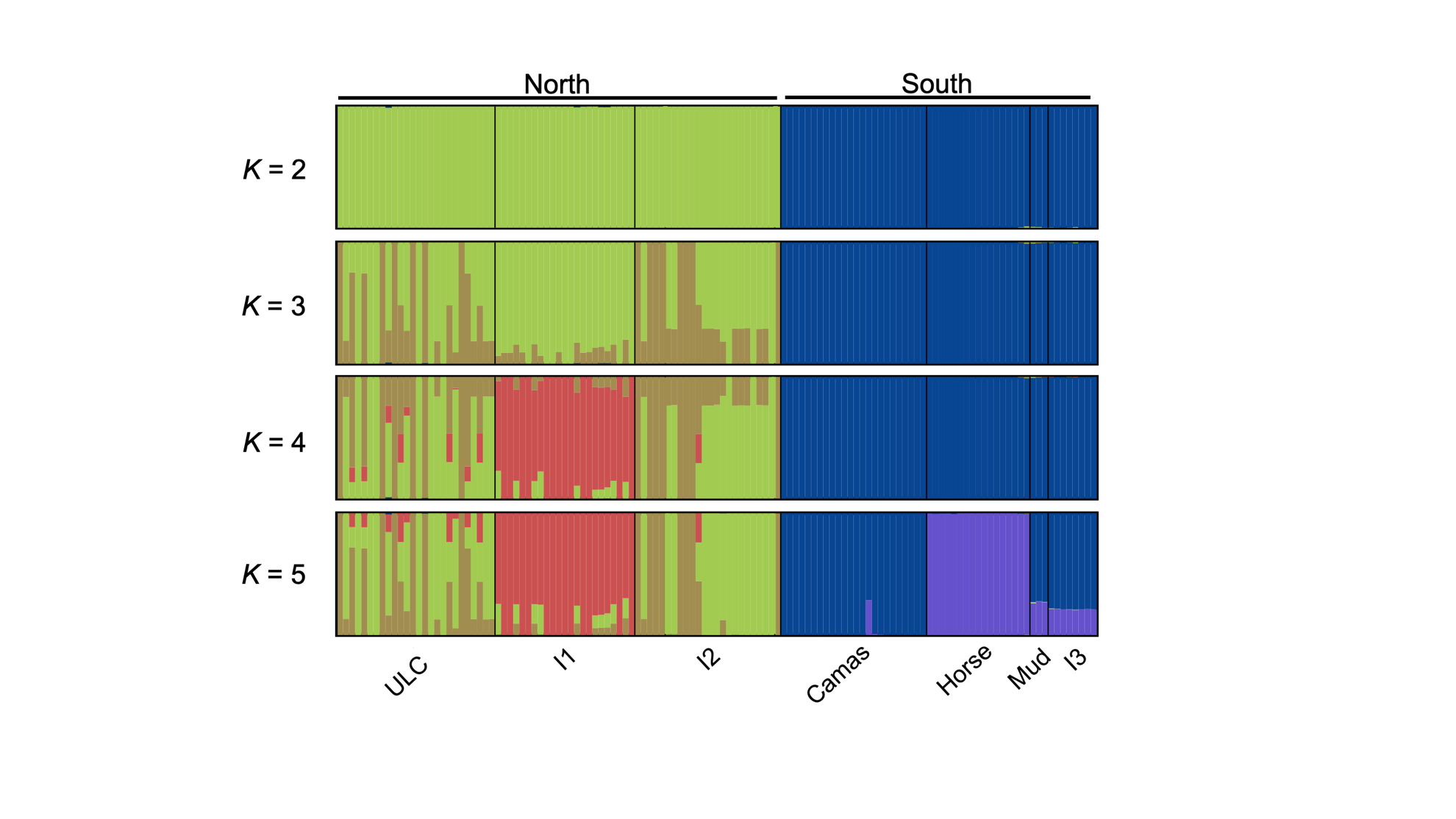
**Figure S4**. structure results for analyses of *K*-values 2 – 5. Population designations are noted along the top of *K* = 2 and sites names (as displayed in Fig. 1) are shown across the bottom of *K* = 5. Solid vertical black lines delineate sampling sites. ULC = Upper Ladd Canyon.

**Figure S5**. Principal component analyses including only non-clonal *P. oregonus* individuals. Colors and shapes are consistent with Fig. 3. Variation explained by PC axes are provided in parentheses.


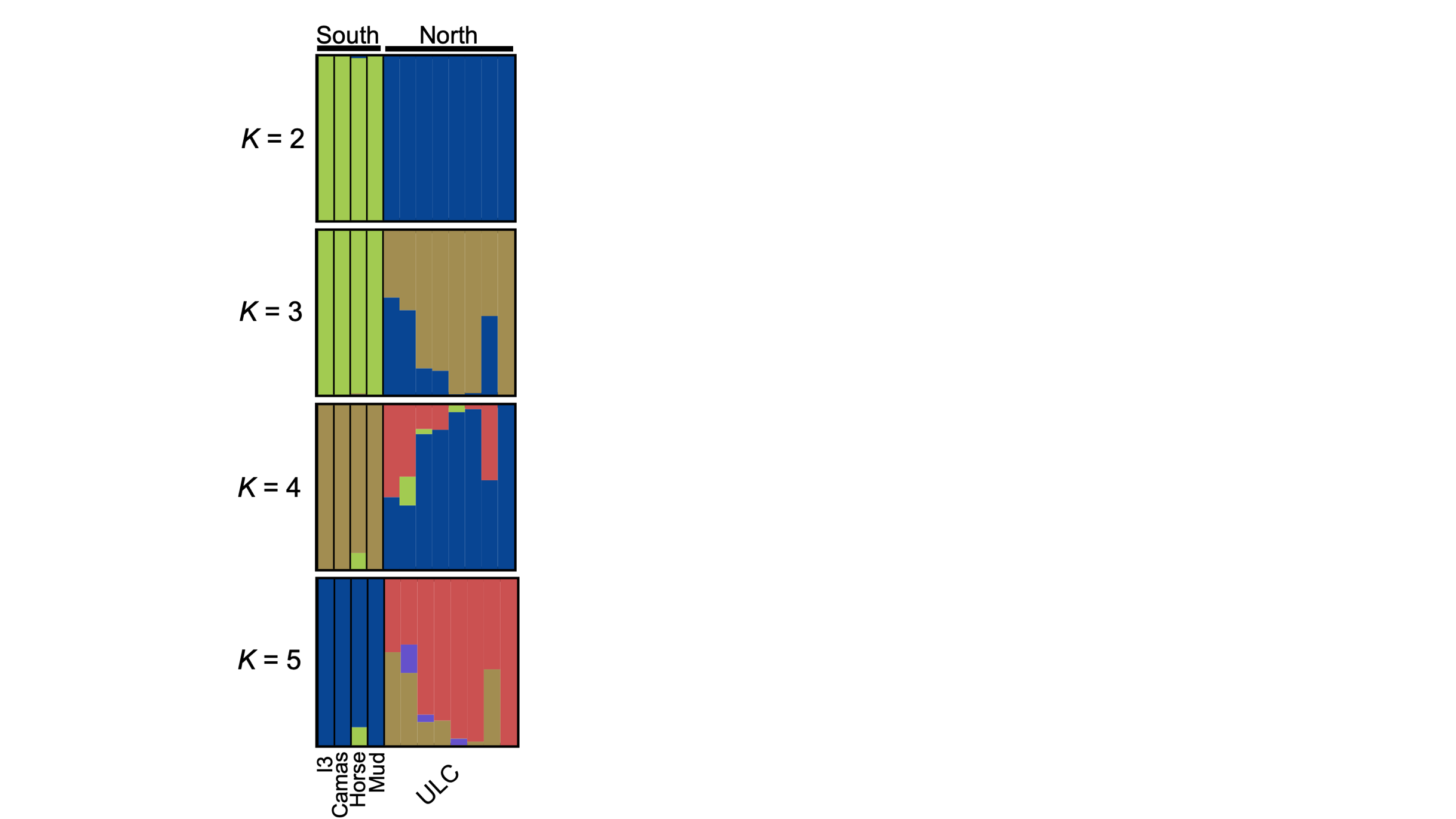


**Figure S6**. structure results for analyses of *K*-values 2 – 5 using only individuals identified as non-clones. Population designations are noted along the top of *K* = 2 and sites names (as displayed in Fig. 1) are shown across the bottom of *K* = 5. Solid vertical black lines delineate sampling sites. ULC = Upper Ladd Canyon.
